# Recurrent Calciphylaxis With Discordant Parathyroid Hormone Trajectory in a Patient With HIV-Associated End-Stage Renal Disease: A Case Report

**DOI:** 10.7759/cureus.109881

**Published:** 2026-05-29

**Authors:** Adnan Asad, Issa Jundi, Wafa Asad, Charles Bonanno

**Affiliations:** 1 Medicine and Surgery, Nova Southeastern University Kiran C. Patel College of Osteopathic Medicine, Fort Lauderdale, USA; 2 Internal Medicine, Memorial Healthcare, Pembroke Pines, USA; 3 Internal Medicine, Nova Southeastern University, Fort Lauderdale, USA; 4 Nephrology, Memorial Healthcare, Pembroke Pines, USA

**Keywords:** calciphylaxis, complications related to calciphylaxis treatment, end stage renal disease (esrd), hiv aids, pth

## Abstract

Calciphylaxis, also termed calcific uremic arteriolopathy, is a rare and devastating complication of end-stage renal disease (ESRD). Its occurrence in patients with HIV-associated ESRD is sparsely characterized in the literature.

We report a 36-year-old African American woman with ESRD secondary to longstanding hypertension and virologically suppressed HIV infection (CD4 count 348 cells/μL) who developed three discrete presentations of recurrent calciphylaxis over 18 months, with histopathologic confirmation at initial diagnosis and at first recurrence. At initial presentation (T1), intact parathyroid hormone (iPTH) was 806.5 pg/mL; she was treated with paricalcitol, sevelamer carbonate, and intravenous sodium thiosulfate (STS) but remained intermittently nonadherent to hemodialysis and prescribed therapies, with a single-pool Kt/V of 0.29 documented at recurrence. At second presentation 12 months later (T2), iPTH was 308.4 pg/mL despite active cutaneous lesions; management included surgical debridement, broad-spectrum antibiotics for superimposed polymicrobial soft tissue infection, continued STS, and dialysis intensification. Six months after T2 (T3), the patient represented with lesion expansion and new ulcerations, and iPTH had risen to 1,764.9 pg/mL alongside hypocalcemia and hyperphosphatemia, consistent with progression toward tertiary hyperparathyroidism. Parathyroidectomy was deferred at T2 and T3 because of acute infectious burden and uncontrolled hyperkalemia, respectively.

This case illustrates the diagnostic and management complexity of recurrent calciphylaxis in HIV-associated ESRD, demonstrates how intermittent treatment adherence may produce highly discordant iPTH measurements that complicate threshold-based monitoring, and underscores the importance of multidisciplinary planning before surgical and metabolic barriers to parathyroidectomy accumulate.

## Introduction

Calciphylaxis, also termed calcific uremic arteriolopathy, is a rare but devastating complication of end-stage renal disease (ESRD) characterized by progressive medial vascular calcification, thrombosis, and occlusion of small- to medium-sized arterioles within dermal and subcutaneous tissue. It affects an estimated 1-4% of patients on maintenance hemodialysis and carries a one-year mortality exceeding 50% [1-3]. Recognized risk factors include female sex, obesity, hypoalbuminemia, warfarin exposure, and elevated calcium-phosphate product [1,2]. Proposed mechanisms include dysregulated calcium-phosphate homeostasis, secondary hyperparathyroidism, vascular smooth muscle cell osteogenic transformation, endothelial dysfunction, and impaired activity of matrix Gla protein, a potent endogenous inhibitor of vascular calcification [1-4]. Calciphylaxis may occur in the absence of overt hypercalcemia or hyperphosphatemia, suggesting that intact parathyroid hormone (iPTH)-driven bone turnover and cumulative vascular injury may act as independent pathogenic drivers [1,2].

Calciphylaxis in patients with HIV-associated ESRD is sparsely described in the published literature, despite a growing population of HIV-positive individuals progressing to ESRD on long-term antiretroviral therapy. Whether HIV-related immune activation, endothelial dysfunction, or specific antiretroviral exposures meaningfully modify mineral metabolism and vascular calcification risk in this population remains incompletely understood.

Management of calciphylaxis remains heterogeneous and is largely informed by observational data. Recurrence affects approximately 14% of survivors and is associated with substantially higher morbidity from cumulative tissue necrosis and superimposed infection [5]. Current approaches emphasize wound care, mineral metabolism optimization, sodium thiosulfate (STS), and dialysis intensification [1-3]. Parathyroidectomy has been proposed for refractory or recurrent disease in patients with uncontrolled secondary hyperparathyroidism, although timing and patient selection require individualization [6-8]. We describe a case of recurrent, biopsy-confirmed calciphylaxis across three presentations in a young woman with HIV-associated ESRD, illustrating the diagnostic and management complexity introduced by treatment nonadherence and discordant biochemical findings.

## Case presentation

A 36-year-old African American woman with ESRD secondary to longstanding hypertension and virologically suppressed HIV infection (CD4 count 348 cells/μL, undetectable viral load on antiretroviral therapy) was first diagnosed with biopsy-confirmed calciphylaxis at initial presentation (T1) of the left medial thigh, at which time iPTH was 806.5 pg/mL. She was managed conservatively with paricalcitol 1 mcg daily, sevelamer carbonate 800 mg three times daily, and intravenous STS 25 g at each hemodialysis session. Despite this regimen, she remained intermittently nonadherent to hemodialysis and prescribed therapies. No anticoagulation, including warfarin, had been prescribed at any prior encounter. Contemporaneous mineral metabolism laboratory values from T1 beyond iPTH were not retrievable at the time of recurrence, representing a limitation of the chronological reconstruction.

At second presentation (T2), 12 months after T1, she presented with a four-day history of progressively worsening left upper and lower extremity pain with reduced range of motion and difficulty ambulating. Review of dialysis records confirmed ongoing suboptimal adherence, with a single-pool Kt/V of 0.29, far below the recommended minimum of 1.2, indicating severe dialysis inadequacy and likely contributing to persistent metabolic derangement. Physical examination revealed a painful, indurated, hyperpigmented plaque with central ulceration along the left medial thigh measuring 9.8 × 10.7 cm (depth 0.8 cm), a second ulcerating lesion with exposed dermis in the left axillary region, and surrounding livedo racemosa, a reticular violaceous pattern reflecting arteriolar occlusion consistent with the underlying vasculopathy. Distal pulses were intact bilaterally. Laboratory evaluation demonstrated serum calcium 8.9 mg/dL (albumin-corrected 9.8 mg/dL), phosphorus 6.2 mg/dL, albumin 2.4 g/dL, alkaline phosphatase 261 IU/L, 25-hydroxyvitamin D 16.8 ng/mL, blood urea nitrogen 24 mg/dL, creatinine 5.7 mg/dL with an estimated glomerular filtration rate (eGFR) of 9 mL/min/1.73 m², and serum potassium 5.5 mmol/L. Admission iPTH was 308.4 pg/mL, substantially lower than at T1, but still consistent with persistent secondary hyperparathyroidism in the context of active cutaneous disease (Table 1).

Antiphospholipid antibody panel was negative. The differential diagnosis included oxalate vasculopathy, cholesterol embolization syndrome, and warfarin-induced skin necrosis; the latter was excluded by the absence of any prior anticoagulant exposure [1,2]. Empiric ampicillin-sulbactam was initiated for superimposed soft tissue infection; wound cultures grew mixed gram-positive cocci, gram-negative bacilli, and gram-positive bacilli, and antibiotic therapy was escalated to vancomycin and ampicillin-sulbactam. Surgical debridement was performed under general anesthesia; histopathologic examination confirmed calcification of small- and medium-sized subcutaneous arteries with associated inflammatory infiltrates and intimal fibrosis, consistent with calciphylaxis [1,2]. Parathyroidectomy was considered but deferred given the acute infectious burden and hemodynamic instability. She was discharged with adjunctive hyperbaric oxygen therapy three times weekly, with which she was subsequently noncompliant.

At third presentation (T3), six months after T2 and 18 months after T1, she presented with expansion of prior lesions and new ulcerations on the left heel and digits, in the setting of continued nonadherence to hemodialysis and prescribed therapies. Repeat iPTH had risen markedly to 1,764.9 pg/mL alongside serum calcium 7.5 mg/dL, phosphorus 6.2 mg/dL, albumin 4.1 g/dL, alkaline phosphatase 111 IU/L, 25-hydroxyvitamin D 15.4 ng/mL, and creatinine 7.2 mg/dL, a constellation consistent with evolving tertiary hyperparathyroidism in which glandular activity is no longer suppressed by circulating calcium. Serum potassium was 6.6 mmol/L. CT scan of the left lower extremity revealed subcutaneous edema, and extensive atherosclerosis and calcification of the wound site with no underlying involvement of the musculature (Figures 1, 2). Clinical and morphological features were consistent with progression of the previously biopsy-confirmed disease. Parathyroidectomy was again considered but deferred because of operative contraindications including uncontrolled hypertension and hyperkalemia; limited transplant candidacy in the context of persistent nonadherence further informed surgical planning. Bedside wound debridement was performed, broad-spectrum antibiotics were continued, sodium zirconium cyclosilicate 10 g daily was added for hyperkalemia, and cinacalcet 90 mg orally once daily was initiated for ongoing secondary hyperparathyroidism management. Throughout the 18-month course, social work referrals, case management consultations, and documented interdisciplinary team discussions were engaged at each hospitalization to address barriers to hemodialysis adherence and medication compliance. A behavioral health referral was placed; however, the patient did not maintain follow-up, citing concern that attending a shared clinical setting might compromise her privacy among acquaintances. The patient was discharged with intensified adherence counseling and outpatient nephrology follow-up (Tables 1, 2). 

**Figure 1:**
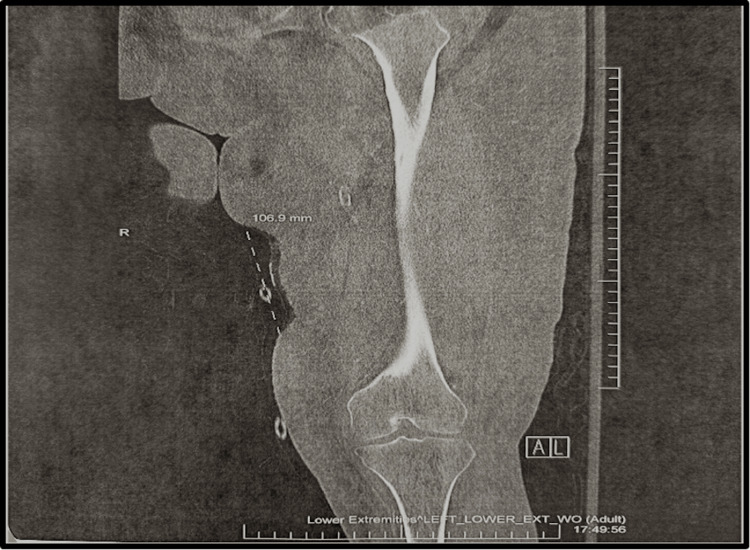
Coronal CT scan of left lower extremity Coronal CT of the left lower extremity demonstrating extensive subcutaneous vascular calcification localized to the area of skin involvement, without extension to the underlying musculature.

**Figure 2:**
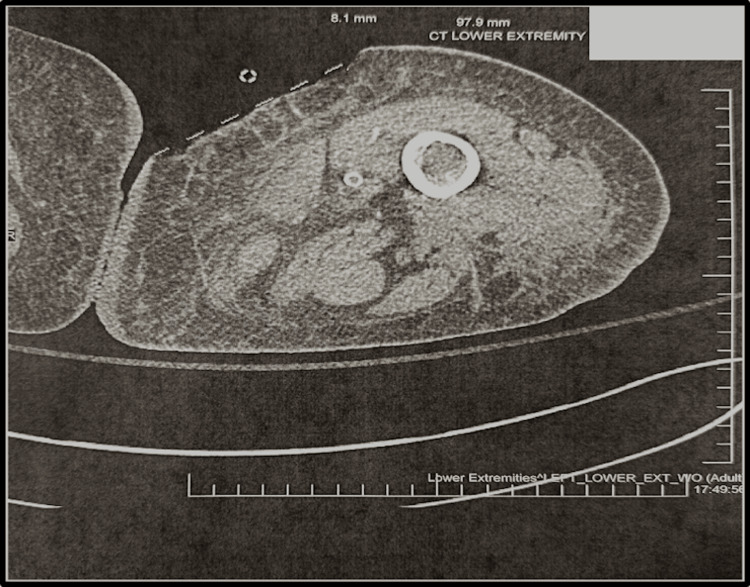
Transverse CT scan of left lower extremity Transverse CT of the left lower extremity at the level of the thigh lesion, demonstrating subcutaneous edema and calcification of subcutaneous arterioles corresponding to the area of biopsy-confirmed disease.

**Table 1:** Clinical timeline of three presentations of recurrent calciphylaxis over an 18-month course iPTH: Intact parathyroid hormone; STS: Sodium thiosulfate

Timepoint	Time from T1	Key clinical findings	Key interventions
T1 (initial)	Month 0	Biopsy-confirmed calciphylaxis with cutaneous lesions; iPTH 806.5 pg/mL. At T1, presenting lesions were identified and biopsy obtained on the left medial thigh.	Paricalcitol, sevelamer carbonate, intravenous STS; conservative management. Subsequent intermittent nonadherence to dialysis and pharmacotherapy.
T2	Month 12	Painful indurated plaque with central ulceration on left medial thigh (9.8 × 10.7 cm), axillary ulcer, surrounding livedo racemosa; superimposed polymicrobial soft tissue infection; iPTH 308.4 pg/mL; single-pool Kt/V 0.29.	Surgical debridement; vancomycin and ampicillin-sulbactam; continued STS; dialysis intensification; hyperbaric oxygen therapy on discharge. Parathyroidectomy deferred (acute infectious burden, hemodynamic instability).
T3	Month 18	Expansion of prior lesions; new ulcerations on left heel and digits; iPTH 1,764.9 pg/mL with hypocalcemia (7.5 mg/dL) and hyperphosphatemia (6.2 mg/dL), consistent with evolving tertiary physiology; hyperkalemia (6.6 mmol/L); uncontrolled hypertension.	Bedside wound debridement; continued broad-spectrum antibiotics; sodium zirconium cyclosilicate for hyperkalemia; adherence counseling and outpatient nephrology follow-up. Parathyroidectomy deferred (uncontrolled hyperkalemia, hypertension; limited transplant candidacy).

**Table 2:** Biochemical parameters at three presentations across an 18-month course of recurrent calciphylaxis iPTH: Intact parathyroid hormone; NR: Not retrievable

Parameter (reference range)	T1 (initial)	T2 (12 months)	T3 (18 months)
iPTH (pg/mL; 15–65)	806.5	308.4	1,764.90
Serum calcium (mg/dL; 8.5–10.5)	NR	8.9	7.5
Albumin-corrected calcium (mg/dL)	NR	9.8	7.5
Phosphorus (mg/dL; 2.5–4.5)	NR	6.2	6.2
Albumin (g/dL; 3.5–5.0)	NR	2.4	4.1
Alkaline phosphatase (IU/L; 35–104)	NR	261	111
25-hydroxyvitamin D (ng/mL; 30–100)	NR	16.8	15.4
Serum creatinine (mg/dL)	NR	5.7	7.2
Serum potassium (mmol/L; 3.5–5.0)	NR	5.5	6.6
Single-pool Kt/V (target ≥ 1.2)	NR	0.29	NR

## Discussion

Recurrent calciphylaxis is a clinically devastating complication of ESRD with limited high-quality evidence to guide longitudinal monitoring or the timing of surgical intervention. This case illustrates several features of the disease that complicate routine threshold-based management. The patient's iPTH trajectory across three presentations was strikingly nonmonotonic: 806.5 pg/mL at T1, 308.4 pg/mL at T2, and 1,764.9 pg/mL at T3. Active cutaneous disease was present at all three timepoints, including at T2, when iPTH had transiently fallen well below the value recorded at initial diagnosis. The most important clinical observation from this trajectory is not that iPTH itself functions as a disease-activity biomarker, but rather that isolated iPTH values can be highly misleading in this setting, and that several factors specific to this patient likely contributed to the apparent disconnection between biochemistry and clinical course.

Treatment nonadherence is the most parsimonious driver of the observed iPTH variability. The patient was intermittently nonadherent to hemodialysis throughout the 18-month course, with a single-pool Kt/V of 0.29 documented at T2, below the recommended minimum of 1.2. She was also inconsistent with paricalcitol, sevelamer carbonate, and STS administration. In this context, sequential iPTH values reflect highly variable exposure to therapies known to suppress PTH (vitamin D analogues, phosphate binders, dialysis-mediated phosphate clearance) rather than a stable underlying biological signal. The apparent transient suppression at T2 likely reflects high-turnover bone disease in the context of advanced secondary hyperparathyroidism - alkaline phosphatase was markedly elevated at 261 IU/L, consistent with accelerated bone resorption - rather than true metabolic control. By contrast, the marked rise at T3 in the setting of frank hypocalcemia (serum calcium 7.5 mg/dL) is consistent with progression toward autonomous glandular activity (tertiary physiology), in which PTH secretion is no longer appropriately regulated by circulating calcium [9,10].

At the mechanistic level, sustained PTH elevation drives bone morphogenetic protein-2- and Wnt-mediated signaling pathways that promote osteogenic transdifferentiation of vascular smooth muscle cells independently of serum calcium and phosphate concentrations [9,10]. This provides a plausible biological basis for ongoing vascular calcification even when calcium and phosphorus appear within conventional target ranges. The Kidney Disease: Improving Global Outcomes (KDIGO) chronic kidney disease - mineral and bone disorder guidelines recommend targeting iPTH at approximately two to nine times the upper limit of normal in dialysis patients, with escalating intervention when levels rise progressively [11]. The present case illustrates a key limitation of threshold-based frameworks: in patients with treatment nonadherence and high-turnover bone disease, isolated iPTH values may transiently fall within or near guideline targets despite ongoing pathologic activity, and clinical correlation with cutaneous findings remains essential.

Therapeutic priorities at each presentation in this patient included STS at 25 g intravenously three times weekly at the conclusion of dialysis, which acts via calcium chelation, antioxidant activity, and vasodilation [12]. Surgical debridement for wound management; and antibiotic escalation for superimposed polymicrobial infection. Dialysis intensification is critical for reducing uremic solute burden and improving phosphate clearance [13]. Warfarin, one of the strongest modifiable risk factors for calciphylaxis through impairment of matrix Gla protein carboxylation [1,2], was notably absent from this patient’s medication history, excluding warfarin-induced vascular calcification as a contributing mechanism. Calcimimetics represent a pharmacologic escalation step in secondary hyperparathyroidism management that warrants specific attention in this case. Cinacalcet, an oral allosteric activator of the calcium-sensing receptor, and etelcalcetide, an intravenous calcimimetic administered at the conclusion of each hemodialysis session, both reduce iPTH, calcium-phosphate product, and fibroblast growth factor-23 in maintenance dialysis patients [11]. Etelcalcetide carries a practical advantage in patients with inconsistent oral medication adherence because drug delivery does not depend on patient-initiated dosing. In this patient, calcimimetic therapy was not documented at T1 or T2 despite severe secondary hyperparathyroidism at both presentations; no explicit clinical rationale for this delay was identified in the available records. Cinacalcet 90 mg daily was first initiated at T3, when iPTH had already risen to 1,764.9 pg/mL. Given the patient’s established pattern of oral medication nonadherence throughout the course, earlier consideration of etelcalcetide as the preferred formulation may have offered more reliable PTH suppression and potentially modified the trajectory leading to the third presentation.

Parathyroidectomy has been associated with improved wound healing and prolonged survival in retrospective series of calciphylaxis patients with refractory secondary hyperparathyroidism [6-8]. Lal et al. identified parathyroidectomy as an independent predictor of improved survival on multivariate analysis [7], and a recent nationwide study confirmed elevated perioperative morbidity specifically among calciphylaxis patients, underscoring the need for careful patient selection [14]. Parathyroidectomy carries substantial perioperative risk, including hungry bone syndrome and protracted hypocalcemia [15]. Subtotal parathyroidectomy may reduce the depth of postoperative hypocalcemia, while total parathyroidectomy without autotransplantation may be preferable in patients with limited transplant candidacy in whom exogenous PTH supplementation is feasible [16]. In this patient, persistent nonadherence, recurrent infection, and hyperkalemia precluded timely surgical intervention; limited transplant candidacy further informed the multidisciplinary approach. The present case underscores how surgical, infectious, and adherence-related barriers can compound to delay potentially beneficial intervention. Multidisciplinary engagement including nephrology, endocrine surgery, infectious diseases, wound care, and palliative care initiated early in the disease course is essential. In this patient, despite repeated social work referrals, case management consultations, and documented interdisciplinary team discussions across all three hospitalizations, adherence to hemodialysis and prescribed therapies remained poor throughout the course. The patient’s non-engagement with behavioral health services, attributed to privacy concerns in a shared clinical setting, underscores the importance of identifying patient-specific barriers to psychosocial support rather than treating nonadherence as a uniform behavioral failure. Exploring individualized options such as telehealth-based mental health services, patient-specific scheduling, or community health worker engagement may better address stigma-related barriers in this population.

Calciphylaxis in HIV-associated ESRD is sparsely described, and the present case adds to the limited literature on this comorbid presentation. HIV infection is associated with chronic immune activation, endothelial dysfunction, and accelerated vascular disease that persist despite virologic suppression on antiretroviral therapy [17], all of which may plausibly intersect with the vascular calcification pathways implicated in calciphylaxis. Tenofovir-based antiretroviral regimens have been linked to proximal tubular dysfunction with renal phosphate wasting and, in severe cases, Fanconi syndrome, which may contribute to mineral metabolism perturbations independent of conventional ESRD-related secondary hyperparathyroidism [18]. Whether such mechanisms meaningfully modify calciphylaxis risk or clinical course in HIV-positive patients with advanced kidney disease remains an open question, and observational studies in larger HIV-positive ESRD populations are needed.

Several limitations should be acknowledged. First, this is a single-patient observation and does not establish a generalizable framework for monitoring or surgical timing in calciphylaxis. Second, profound and intermittent treatment nonadherence is the dominant likely driver of the observed iPTH variability. In adherent patients, the biochemical trajectory might appear quite different. Third, contemporaneous mineral metabolism values at T1 beyond iPTH were not retrievable, limiting full chronological reconstruction. Fourth, while clinical and histopathologic features at T1 and T2 confirmed calciphylaxis, the third presentation was characterized clinically and morphologically without repeat biopsy. Finally, the specific contribution of HIV-associated mechanisms in this patient cannot be determined from a single case and is included as a hypothesis-generating consideration.

## Conclusions

Recurrent calciphylaxis in patients with HIV-associated ESRD complicated by treatment nonadherence presents substantial diagnostic and therapeutic challenges. This case illustrates how isolated iPTH values can mislead clinical decision-making when adherence to dialysis and pharmacotherapy is highly variable, and underscores that clinical correlation with cutaneous findings must remain central to longitudinal monitoring rather than reliance on biochemical thresholds alone. Calcimimetic therapy, particularly intravenous etelcalcetide in patients with adherence challenges, represents a pharmacologic intervention that should be optimized as part of the escalation pathway before or alongside surgical planning. Timely multidisciplinary planning, including consideration of parathyroidectomy before infectious, hemodynamic, and adherence-related barriers accumulate, may improve outcomes in selected patients. Further study of calciphylaxis in HIV-associated ESRD is warranted given the increasing prevalence of HIV-positive individuals progressing to long-term renal replacement therapy.
